# An in-vivo pilot study into the effects of FDG-mNP in cancer in mice

**DOI:** 10.1371/journal.pone.0202482

**Published:** 2018-08-20

**Authors:** Omer Aras, Gillian Pearce, Adam J. Watkins, Fuad Nurili, Emin Ilker Medine, Ozge Kozgus Guldu, Volkan Tekin, Julian Wong, Xianghong Ma, Richard Ting, Perihan Unak, Oguz Akin

**Affiliations:** 1 Department of Radiology, Memorial Sloan Kettering Cancer Center, New York, NY, United States of America; 2 School of Engineering and Applied Sciences, Aston University, Birmingham, United Kingdom; 3 Aston Research Centre for Healthy Ageing, School of Life and Health Sciences, Aston University, Birmingham, United Kingdom; 4 Division of Child Health, Obstetrics and Gynaecology, School of Medicine, Queen's Medical Centre, University of Nottingham, Nottingham, United Kingdom; 5 Department of Nuclear Applications, Institute of Nuclear Sciences, Ege University, Izmir, Turkey; 6 Division of Vascular & Endovascular Surgery, Department of Cardiac, Thoracic & Vascular Surgery, National University Heart Centre, Singapore, Singapore; 7 Molecular Imaging Innovations Institute (MI3), Department of Radiology, Weill Cornell Medicine, New York, New York, United States of America; Brandeis University, UNITED STATES

## Abstract

**Purpose:**

Previously, fluorodeoxy glucose conjugated magnetite nanoparticles (FDG-mNPs) injected into cancer cells in conjunction with the application of magnetic hyperthermia have shown promise in new FDG-mNPs applications. The aim of this study was to determine potential toxic or unwanted effects involving both tumour cells and normal tissue in other organs when FDG-mNPs are administered intravenously or intratumourally in mice.

**Materials and methods:**

FDG-mNPs were synthesized. A group of six prostate-tumour bearing mice were injected with 23.42 mg/ml FDG-mNPs (intravenous injection, n = 3; intratumoural injection into the prostate tumour, n = 3). Mice were euthanized and histological sampling of tissue was conducted for the prostate tumour, as well as for lungs, lymph nodes, liver, kidneys, spleen, and brain, at 1 hour (n = 2) and 7 days (n = 4) post-injection. A second group of two normal (non-cancerous) mice received the same injection intravenously into the tail vein and were euthanised at 3 and 6 months post-injection, respectively, to investigate if FDG-mNPs remained in organs at those time points.

**Results:**

In prostate-tumour bearing mice, FDG-mNPs concentrated in the prostate tumour, while relatively small amounts were found in the organs of other tissues, particularly the spleen and the liver; FDG-mNP concentrations decreased over time in all tissues. In normal mice, no detrimental effects were found in either mouse at 3 or 6 months.

**Conclusion:**

Intravenous or intratumoural FDG-mNPs can be safely administered for effective cancer cell destruction. Further research on the clinical utility of FDG-mNPs will be conducted by applying hyperthermia in conjunction with FDG-mNPs in mice.

## Introduction

The global cancer burden is growing and cancer now causes more deaths than all coronary heart disease or all stroke. In 2012 there were 14.1 million newly diagnosed cancer cases (excluding non-melanoma skin cancer) worldwide with 8.2 million cancer-related deaths [1]. In spite of many years of research at high financial cost, a definitive treatment or cure for cancer presently remains beyond our reach. Although treatments have been developed for cancer, e.g., radiotherapy and chemotherapy, these are not without their problems and patients continue to suffer from detrimental side effects. Many of these side effects arise on account of the highly toxic nature of the chemotherapeutic agents used in the course of the cancer treatment. It has also been established that in the course of time, cancer cells could develop resistance to chemotherapeutic agents used in the treatment of cancer [2, 3]. Furthermore, some types of cancer appear to remain incurable with current radiotherapy and chemotherapy approaches [4]. Clearly, there is scope and potential for new approaches to the treatment of cancer.

Cancer cells all appear to have one distinguishing feature, namely a higher metabolic rate. On account of their fast reproductive rate, they consume glucose at a higher rate than normal cells. This phenomenon is known as the Warburg effect. Cancer cells have a higher rate of glucose uptake with respect to normal cells via the mechanism of glycolysis, which results in the production of adenosine triphosphate through the process of oxidative phosphorylation. This effect was lately demonstrated in many cancer types including neuroblastoma [5].

Previous studies including experimental kinds have involved the investigation of the efficacy of magnetic nanoparticles (mNPs) used in conjunction with chemotherapeutic drugs [6, 7]; several have also studied the effect of this approach on cancer destruction when hyperthermia is applied, showing higher efficacy depending on the temperature and duration of heating [8]. This phenomenon is known as magnetic fluid hyperthermia, whereby the sensitisation of the cancer cells prior to radiotherapy may increase cancer destruction [9–11]. These previous studies frequently involved the use of iron oxide containing mNPs on account of their biocompatibility and low toxicity properties [9–13]. More recent studies have involved the application of radiofrequency (RF) fields to magnetic nanoparticles that are bound on membrane receptors. RF fields have been associated with dissipation of heat via mNPs, facilitating the destruction of tumour cells. The results indicate that cellular signalling mechanisms are involved in cell apoptosis when the temperature of the cancer cell reaches 42°C [14, 15].

In one of our previous in vitro studies [12], we synthesized fludeoxyglucose (FDG) conjugated with iron oxide particles specifically as a Positron Emission Tomography-Magnetic Resonance Imaging (PET-MRI) hybrid compound for potential multimodality medical applications. MCF 7 human breast cancer cells in this study had a higher metabolic rate than normal cells; as such they were able to readily take up the glucose analogue present in our FDG-mNPs which produced apoptotic effects on the cancer cells. Recently, another in vitro pilot study we conducted using a neuroblastoma cell line showed that when FDG-mNPs were injected into these cancer cells and when magnetic hyperthermia was applied, 89% of the cancer cells were destroyed [14, 16]. Safe frequencies of hyperthermia for use in human tissues have been long established [5, 17, 18]. Thus, the present study is an in vivo investigation aimed at discovering if there are potentially any toxic or unwanted effects involving both the tumour cells and other organs in mice when FDG-mNPs are injected intravenously or intratumourally, without the application of hyperthermia.

## Materials and methods

### Synthesis of mNPs

**Fig 1:**
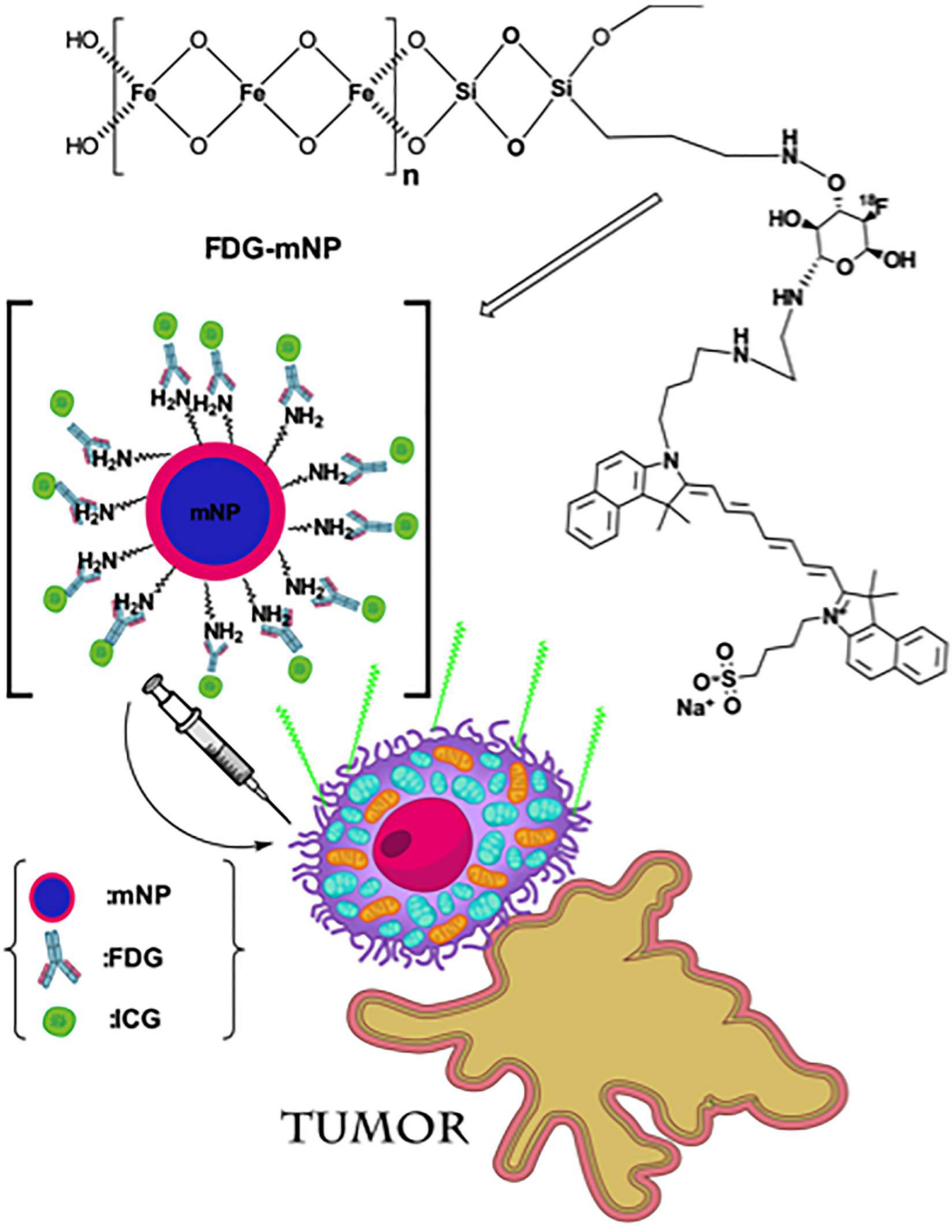
Chemical structure of FDG-mNP and disruption of cancer cell.

Synthesis of mNPs was performed as previously described [12] and chemical structure is shown in Fig 1. Briefly, 2 M FeCl_3_ (Fluka, Istanbul, Turkey) was combined with 80 mM Na_2_SO_3_ (Merck) prior to the addition of 25% NH_3_ solution (Merck) under nitrogen gas. After 30 minutes heating at 70 ^o^C, the particles were washed with a water–ethanol (2:1) mixture and re-suspended in 80% ethanol. The particles were mixed with tetraethyl orthosilicate for 12 hours at 40 ^o^C and washed with methanol prior to incubation with (3-aminopropyl) triethoxysilane (APTES, Sigma, USA) 12 hours at 60 ^o^C with rapid stirring.

Separately, solutions of mannose triflate (Fluka, Istanbul, Turkey) and cysteamine (2-aminoetanethiol; Sigma, Istanbul, Turkey) were prepared in water, mixed heated for 1 hour at 90 ^o^C, precipitated and dried overnight prior to dissolving in dimethyl formamide (Merck). Next, solutions of Kryptofix (Merck), K_2_CO_3_ (Fluka, Istanbul, Turkey) dimethyl formamide (Merkc) and NaF (Merck) were added to 1 ml of the prepared mannose triflate-cysteamine and heated for 20 minutes at 90 ^o^C. The product was purified by sequential passing through a Dowex 50 cation exchange resin column (Sigma, USA), Ambersep 900 quaternary ammonium anion exchange resin (Fluka), Amberlite anion exchange resin (Sigma) and finally a C18 pre-cartridge (Sigma, USA). The purified NaF substituted mannose triflate-cysteamine was mixed with the mNPs prior to the addition of N-Hydroxysuccinimide (Merck) and mixing for 2 hours.

For the labelling of mNPs with indocyanine green (ICG), the mNPs were mixed with carbonyl diimidazole and N-Hydroxysuccinimide for 15 minutes at room temperature prior to the addition of ICG solution (Sigma, USA) and a further 15 minutes mixing at room temperature. Finally, mercaptoethanol (Sigma, USA) was added to the reaction mixture for 2 hours at room temperature prior to washing and storage in phosphate buffer saline (PBS) at 4 ^o^C. ICG labelled FDG-mNPs were checked for excitation and emission spectra at 780 nm 820 nm respectively and eluted in 60% acetonitrile (in distilled water).

### In vivo analysis of ICG-conjugated FDG-mNP tissue distribution

All mice and experimental procedures were conducted using protocols approved by and in accordance with the Weill Cornell Medical Center Institutional Animal Care and Use Committee and were consistent with the recommendations of the American Veterinary Medical Association and the National Institutes of Health Guide for the Care and Use of Laboratory Animals.

Six nude mice (female, weight 20–25 gram) with prostate tumours were used in group 1 by injection of 1.0 × 10^6^ of PC3 cells into right flank region subcutaneously. Tumours were measured weekly until volume exceeded >0.5 cm. Animals were anesthetized with gas isoflurane at 2% concentration mixed with medical grade oxygen. Three mice were injected with FDG-mNPs into the tail vein, and the remaining three were injected with FDG-mNPs intratumourally directly into the prostate tumours itself, all with a concentration of 23.42 mg/ml (10 mg/kg body weight). A biodistribution study of each mouse was performed in vivo by 4.7 T MRI (T2* -4 gradient recalled echo weighted imaging) before and after 1 h and 24 h post injection. Animals were euthanized by CO_2_ asphyxiation after scans were completed. Regions of interest (ROIs) were drawn over the tumour, liver, spleen, right kidney, muscle and lymph node in the right groin using OsiriX software (Pixmeo SARL, Bernex, Switzerland) and images were normalized to the background (muscle) intensity for each mouse at each imaging time points. Samples were also taken from the left kidney, liver, lung, spleen, muscle and brain. The tumour and organs (liver, spleen, lungs, liver, brain, muscle, and lymph node) were immersed fixed in 4% Paraformaldehyde for 24 hours, followed by embedding in paraffin. 5 micron sections were stained for Haemotoxylin and Eosin (H&E) and Prussian blue.

### Quantification of iron staining

The slides were digitally scanned with Pannoramic Flash (3DHistech, Hungary) and relevant tissue areas were exported into tiff format. Quantification of iron staining was performed using ImageJ/FIJI (NIH, Bethesda, Maryland, USA). A colour deconvolution algorithm was used, with RGB vectors for the iron positive areas and counterstain/background stain created from ROIs drawn from example images. Appropriate thresholds were then set for both iron positive regions as well as the tissue area. All iron positive areas were normalized to the total tissue area analyzed per region.

### In vivo MRI

T2* MRI of the mice was carried out on the 300 MHz Bruker 4.7T Biospec scanners (Bruker Biospin MRI GmbH, Ettlingen, Germany) equipped with 640 mT/m ID 115 mm gradient (Resonance Research, Inc., Billerica, MA). RF excitation and acquisition was achieved by a custom-built quadrature birdcage resonator with an inner diameter (ID) of 32 mm (Stark Contrast MRI Coils Research Inc., Erlangen, Germany). The mice were anesthetized with 2% isoflurane (Baxter Healthcare Corp., Deerfield, IL) gas in oxygen. Animal respiration was monitored with a small animal physiological monitoring system (SA Instruments, Inc., Stony Brook, New York, USA). Scout images along three orthogonal orientations were first acquired for animal positioning. We used 3D Multiple Gradient Echo sequence (MGE) to acquire a series of T2*-weighted images with increasing echo time (TE) values 3.3 ms, 7.5ms, 11.4ms and 16.0 ms. Other acquisition parameters were repetition time (TR) 34 ms, field of view (FOV) 30 × 35 × 100 mm with a voxel size of 0.23 × 0.21 × 0.20 mm^3, 3 averages.

### Longitudinal analysis of mNP tissue bio-distribution

All mice and experimental procedures were conducted using protocols approved by, and in accordance with, the UK Home Office Animal (Scientific Procedures) Act 1986 and local ethics committee at Aston University. To determine the tissue persistence of our FDG-mNPs over a prolonged period, we injected two, 3-week-old male NMRI mice intravenously with 8 mg / kg of our FDG-mNPs in 100 μl of sterile PBS. Both mice were maintained on standard chow and water ad libitum for either 3 or 6 months post injection. Both mice showed no visible sign of ill health, were fully mobile and increased or maintained their body weight throughout the study. After 3 or 6 months, mice were culled via cervical dislocation. Heart, kidneys, lungs, liver, spleen, muscle and brain tissue were fixed for 48 hours in 10% neutral buffered formalin (Sigma, UK) at 4^o^ C prior to storage in 70% ethanol at 4^o^ C. Tissues were processed into paraffin wax prior to sectioning at 5 μm. Sections from all tissues were analysed for either for (i) gross morphology by H&E staining (by utilising standard staining protocols) or for (ii) levels of apoptosis using the APO-BrdU TUNNEL Assay Kit (Molecular Probes, Invitrogen, UK). The H&E stained sections were imaged using a CETI Magnum-T microscope connected to a Jenoptik ProgRes CF camera. The APO-BrdU TUNNEL stained sections were imaged on a Leica Microstystems DMI 4000B microscope coupled to a Leica DFC360 FX camera. Fluorescent images were obtained with filters adjusted for nuclear staining with Hoechst and for apoptotic nuclei staining with an Alexa Fluor 448 dye-labelled anti BrdU antibody. Relative tissue staining intensities were measured using Volocity imaging software (Perkin Elmer, USA).

## Results

### Short-term impact of FDG-mNPs

**Fig 2:**
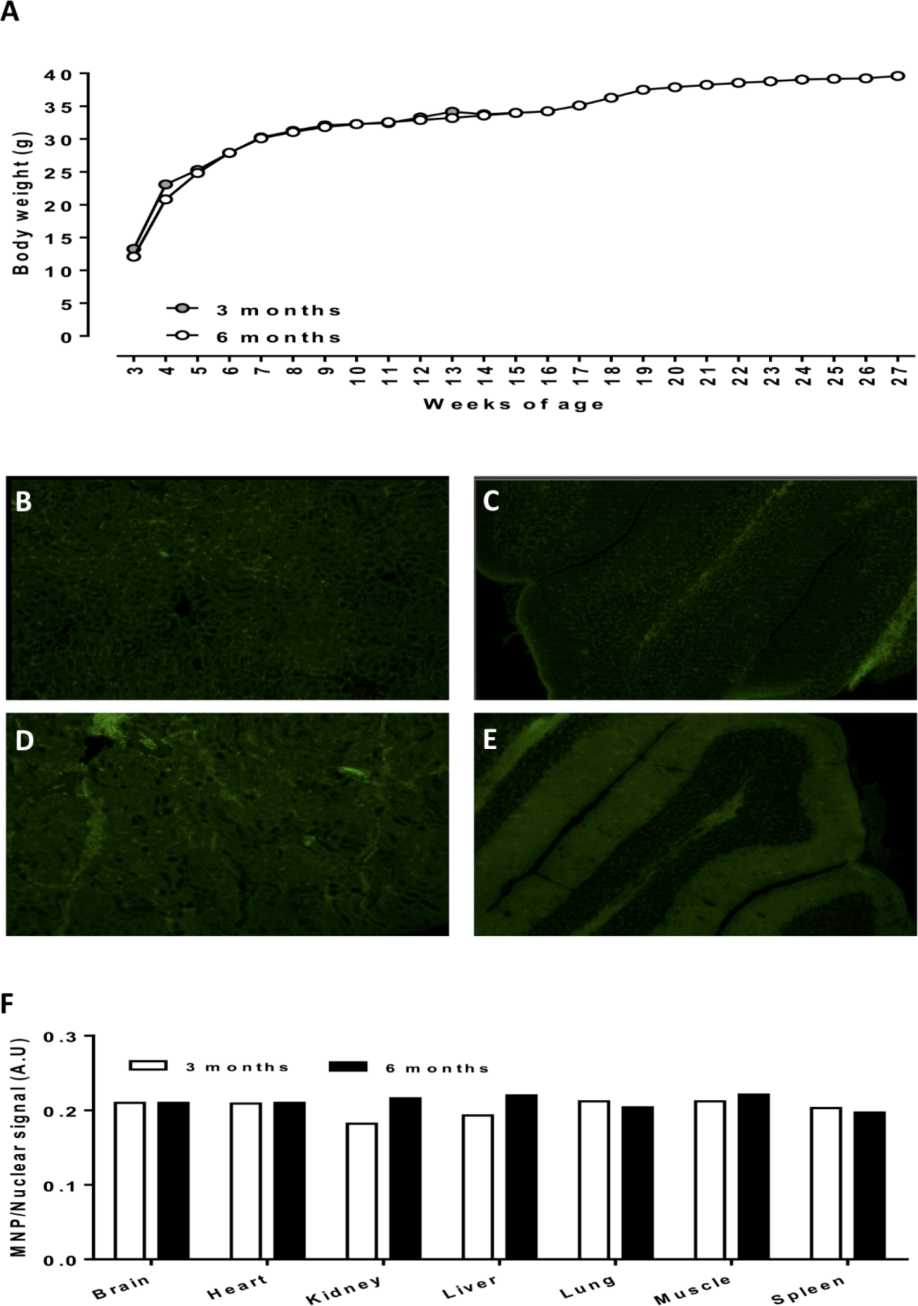
Growth profiles of mice injected at 3 weeks of age with 8mk/kg ICG-conjugated FDG-mNPs (A). Representative fluorescent images of kidney (B, D) and brain (C, E) tissue sections from 3 and 6 month old mice injected with ICG-conjugated FDG-mNPs. Tissue intensity (arbitrary units) staining for ICG-conjugated FDG-mNPs normalised to nuclear Hoechst signal in 3 and 6 month old mice (F). n = 1 mouse per treatment group. Images in B-E are at 10x magnification. FDG: fluorodeoxyglucose; ICG: indocyanine; mNP: magnetic nanoparticle.

**Fig 3:**
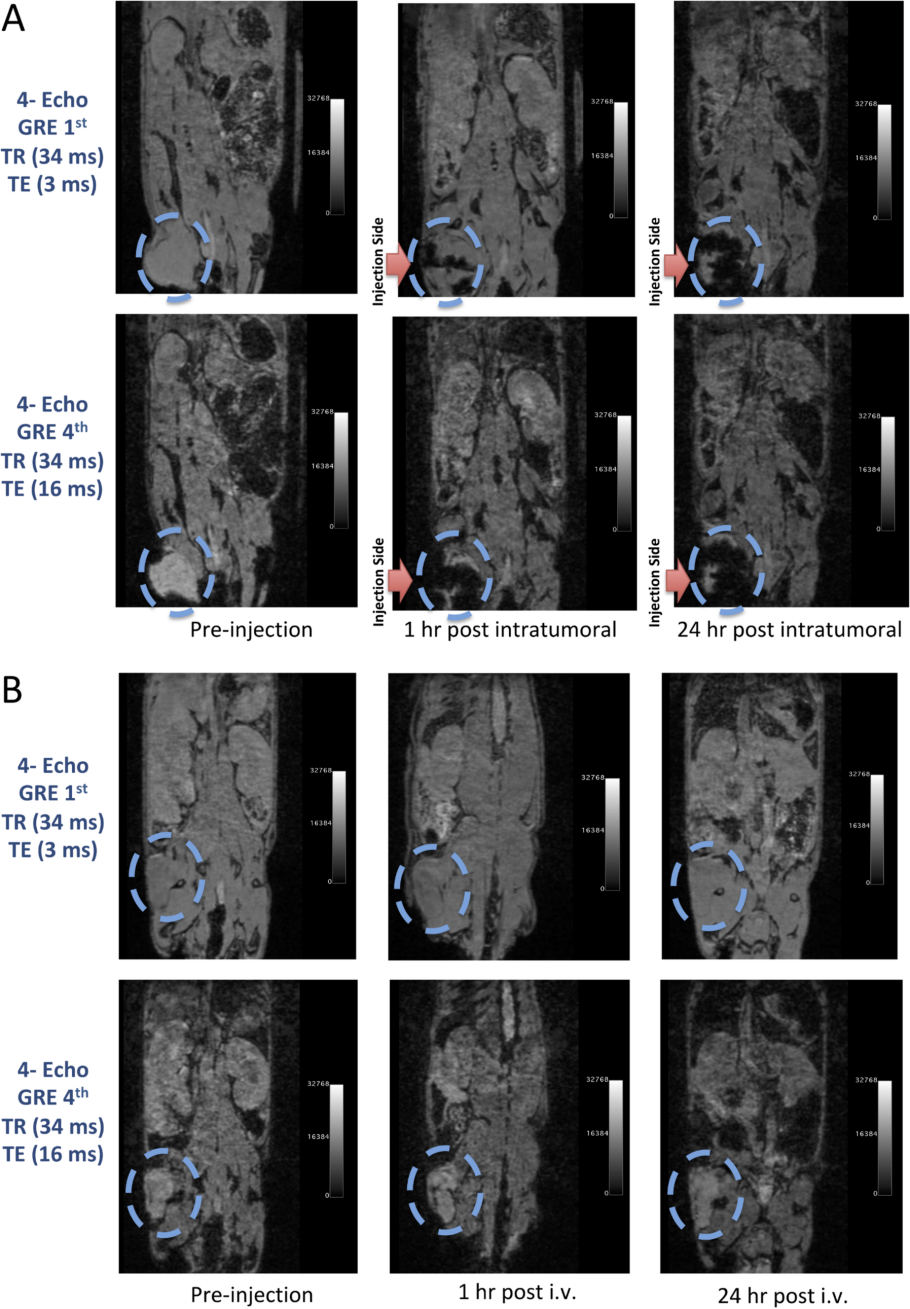
MRI scans of tumorous hind limb of mice before and after intratumoural (*A*) and intravenous (*B*) application of FDG-mNPs. A: The MRI scans of an animal before and after intratumoural injection of FDG-mNPs. T2-weighted gradient echo imaging and multi-echo fast spin echo imaging on a pre-, hour 1^st^ and 24^th^ of injection. B: The MRI scans of an animal before and after intravenous injection of FDG-mNPs. T2-weighted gradient echo imaging and multi-echo fast spin echo imaging on a pre-, hour 1^st^ and 24^th^ of injection. FDG: fluorodeoxyglucose; mNP: magnetic nanoparticle; MRI: magnetic resonance imaging.

Histology results for the mice in group 1, the prostate-cancer bearing mice, are shown in Fig 2. Corresponding MRI images for the mice in group 1 are shown in Fig 3A and 3B. FDG-mNPs injected intratumourally were highly concentrated in the prostate tumour compared with non-cancerous tissues in other organs. One hour post-injection, FDG-mNP concentrations were shown to be high in the tumour itself and the concentration appeared to be lower in the periphery of the tumour compared with more centrally (although we were comparing three mice and such differences could possibly be accounted for by individual variations amongst the mice). Results one hour post-injection also indicated that the concentration of FDG-mNP was greatest in the mice injected intratumourally compared with the mice injected intravenously.

**Fig 4:**
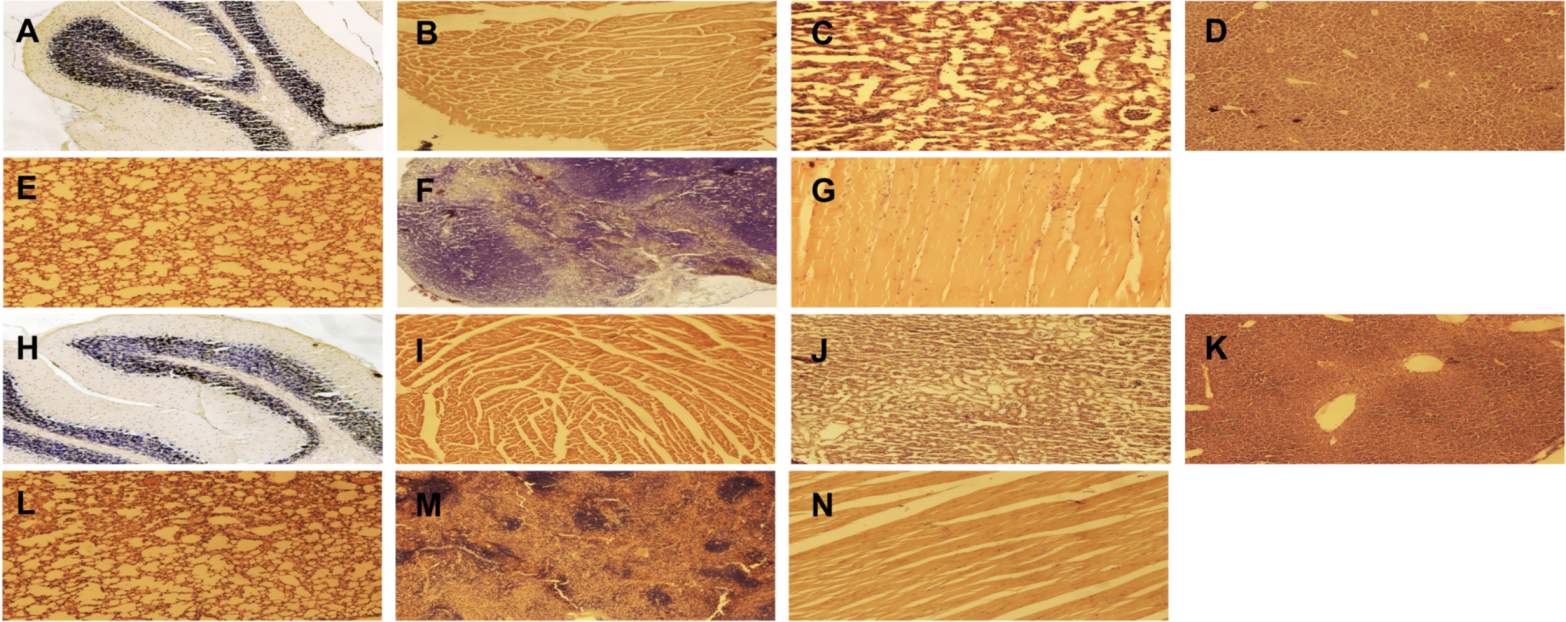
Representative Haemotoxylin and Eosin stained sections from mice injected with ICG-conjugated FDG-mNPs at a concentration of 8mk/kg. Sections show representative images of brain (A), heart (B), kidney (C), liver (D), lung (E), spleen (F) and muscle (G) in a 3 month old mouse. Sections also show representative images of brain (H), heart (I), kidney (J), liver (K), lung (L), spleen (M) and muscle (N) in a 6 month old mouse. All images are at 10x magnification. FDG: fluorodeoxyglucose; ICG: indocyanine; mNP: magnetic nanoparticle.

Histology results for the mice in group 2, the non-cancer containing mice injected intravenously and euthanised at 3 and 6 months, respectively, are shown in Fig 4. We observed no detectable impairment in mouse well-being or behaviour in response to FDG-mNPs in these mice 3 and 6 months post-injection.

**Fig 5:**
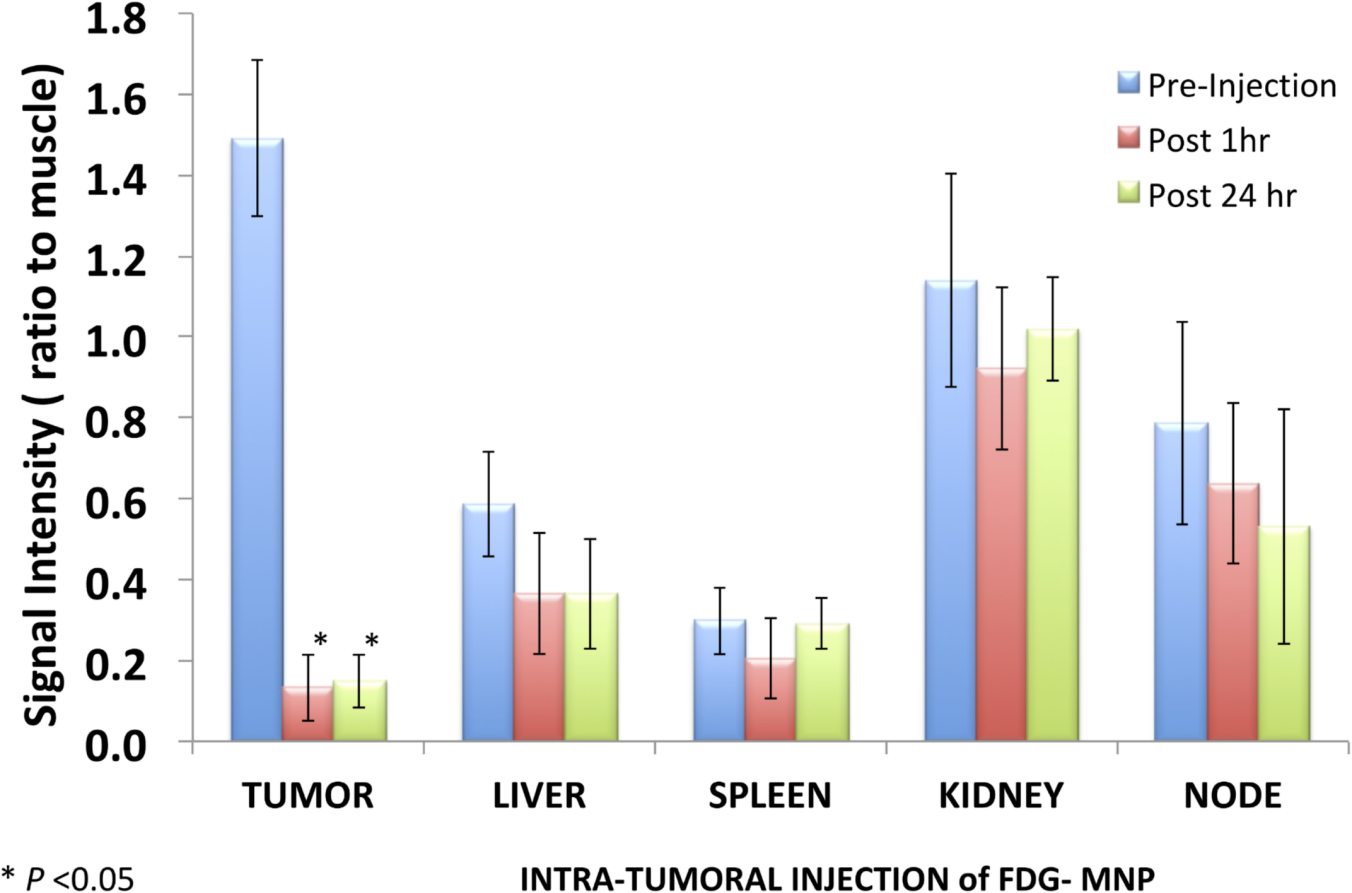
Illustration of signal intensity relative to muscle in tumour and in normal organs (liver, spleen, kidney and node) after intravenous injection of FDG-mNPs. FDG: fluorodeoxyglucose; mNP: magnetic nanoparticle.

**Fig 6:**
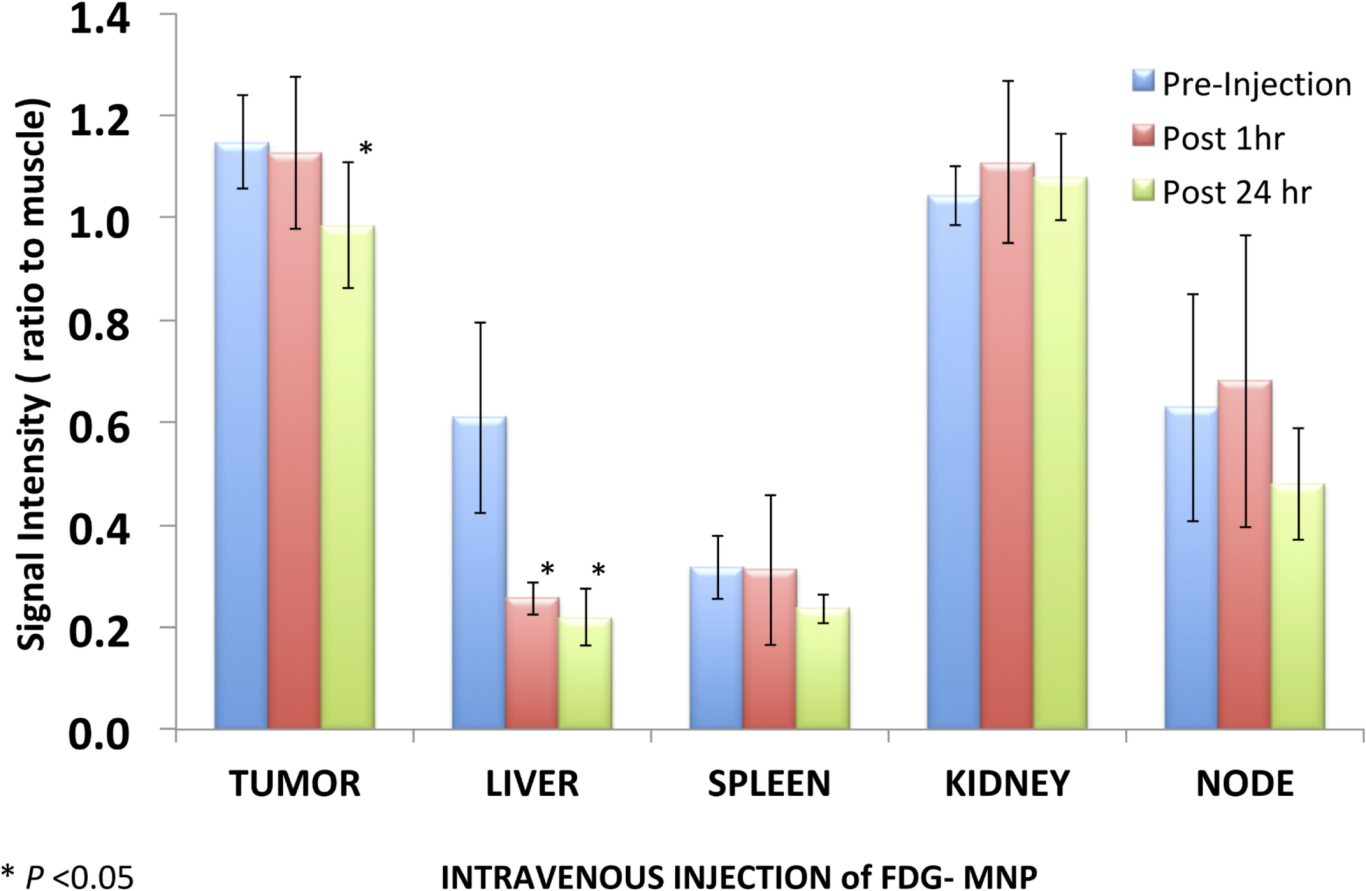
Illustration of signal intensity relative to muscle in tumour and in normal organs (liver, spleen, kidney and node) after intratumoural of FDG-mNPs. FDG: fluorodeoxyglucose; mNP: magnetic nanoparticle.

**Fig 7:**
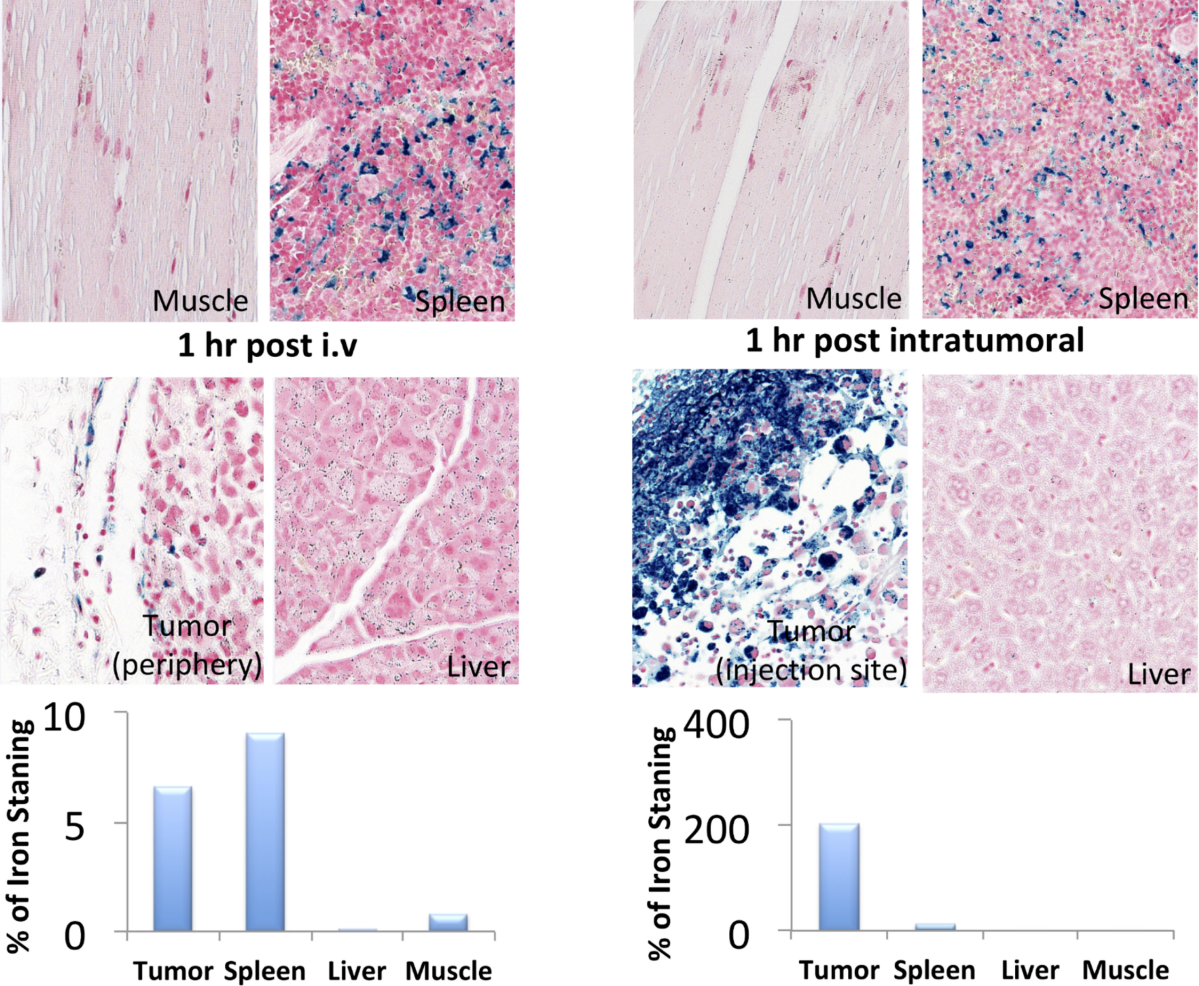
Histological analyses of tumour, muscle, liver and spleen after intratumoural and intravenous injections of FGD-mNPs. FDG: fluorodeoxyglucose; mNP: magnetic nanoparticle.

**Fig 8:**
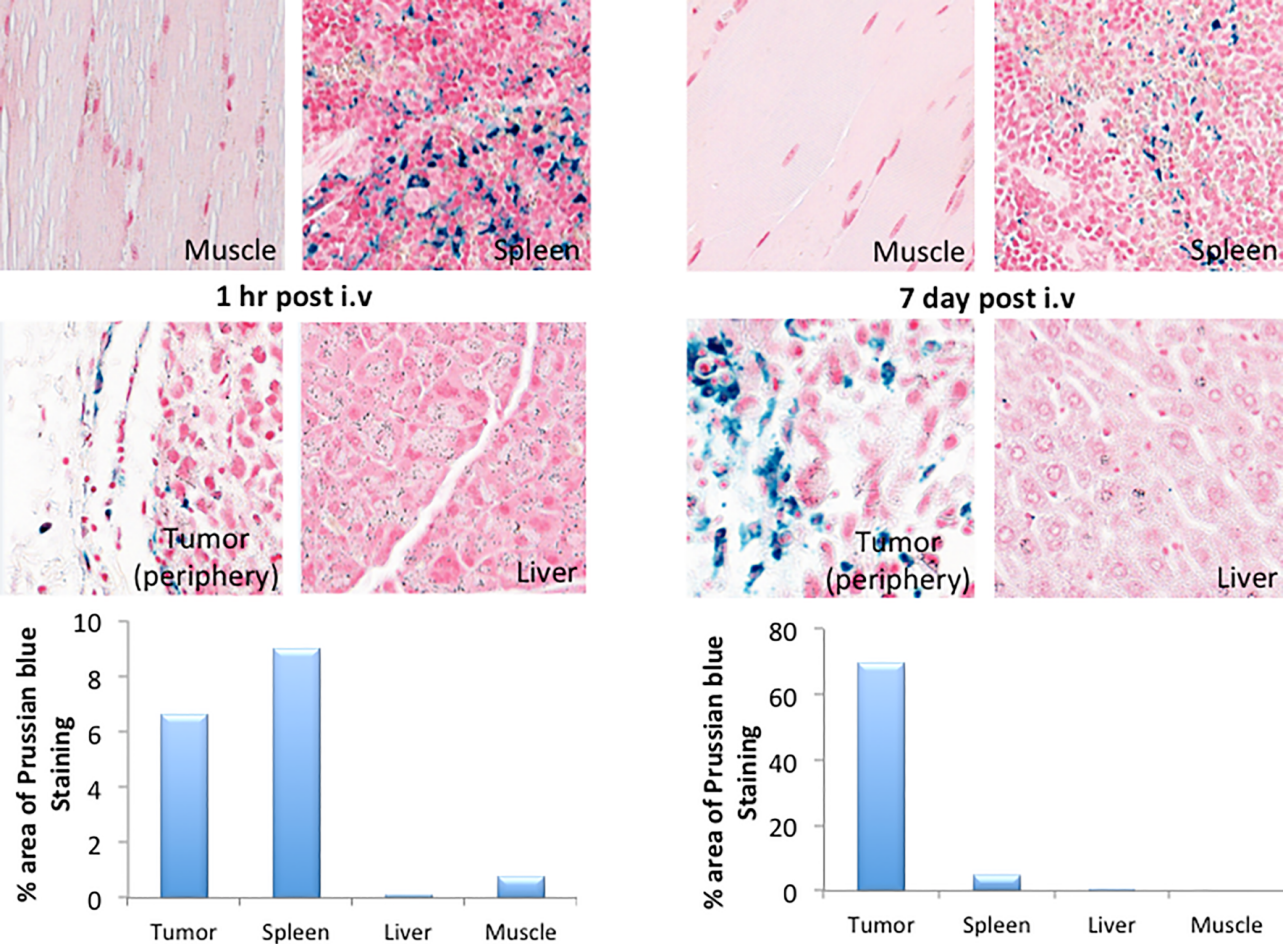
Histological analyses of tumour, muscle, liver and spleen after intravenous injections of FGD-mNPs after 1 hr and 7 days. FDG: fluorodeoxyglucose; mNP: magnetic nanoparticle.

T2-weighted gradient echo imaging and multi-echo fast spin echo imaging showed that FDG-mNPs selectively accumulated in tumours in prostate-tumour bearing mice, as evidenced by a reduction in T2 values and signal decrease in T2-weighted images in various areas of the tumour mass (s 5 and 6). ROI analysis of the MRI signal change in the tumour mass showed approximately ≈11-fold signal reduction in mice receiving intratumoral FDG-mNPs when compared against contralateral muscle background tissue (Fig 5). In addition to our observing MRI signal reduction in the tumour mass, we also observed decreases in MRI signals in the liver and spleen of mice injected with FDG-mNPs because of an iron oxide particle-induced T2 effect. However, the reduction in MRI signal was lower in mice that received intravenous FDG-mNPs compared with mice that received intratumoural FDG-mNPs (≈ 3% in the liver), suggesting that the liver uptake of the nanoparticles was probably a normal biodistribution of FDG-mNPs (Fig 6). To further confirm the distribution of FDG-mNPs in normal and tumour tissues, Prussian blue staining was done on tissue sections obtained from these mice that received FDG-mNPs intravenously or intratumourally (Fig 7). Our results showed FDG-mNPs (labelled with the blue dye) were taken up by normal cells in other organs especially in the spleen, but over time this concentration appeared to decrease in these cells (as in the prostate tumour cells also). In mice injected intravenously, most of the FDG-mNPs appeared to have decreased in the tissues of the normal cells at 7 days post-injection; while the FDG-mNPs appeared to concentrate in the spleen, these are relatively small amounts (Fig 8).

### Long-term impact of FGD-mNPs

Throughout the experiment, there was no detectable impairment in mouse wellbeing. Both the 3 and 6 month mice displayed good condition of coat and no recorded weight loss for up to 6 months (Fig 2A). Tissue sections were analysed for levels of retained FDG-mNPs using fluorescent microscopy (Fig 2B–2E). We observed that any detectable staining present within the samples was not above that of background auto-fluorescence and that tissues from both the 3 and 6 month old mice displayed comparable staining intensities when compared with each other (Fig 2F). We also conducted analyses for levels of apoptosis within all tissues samples and observed no significant or differential levels of apoptosis in any of the samples (data not shown). Histological analysis of brain, heart, kidney, liver, lung, muscle and spleen tissues revealed typical morphology and no overt sign of tissue damage or cellular damage (Fig 4).

## Discussion

Our previous in vitro study indicated that magnetic hyperthermia, when applied to an in vitro cell neuroblastoma cancer line injected with FDG-mNPs, could be used to destroy cancer cells [14]. However, since a relatively large concentration of FDG-mNPs was used in that in vitro study, it was necessary to investigate the effects of FDG-mNPs when used without magnetic hyperthermia and when smaller concentrations are used in vivo. Our current study involved mice with prostate cancer and normal mice, all of which were injected with a therapeutic concentration of FDG-mNP corresponding to that which would be used in humans (the concentration was scaled for body mass and blood volume in the mice).

Our results show that FDG-mNPs do little to no damage to the tumour tissue when used alone without magnetic heating. Moreover, further analysis in the prostate-cancer bearing mice shows that there is relatively little FDG-mNPs remaining in the tissues of the liver, lungs or kidneys, muscle or brain of the mice when FDG-mNPs were injected intravenously or intratumourally. The highest concentration of FDG-mNPs appeared to be in the tumour of the mice injected intratumourally, and this concentration decreased over time from 1 hour post-injection to 7 days (when the mice were euthanised). It seems that in some instances the normal spleen takes up FDG-mNP (although this is still a relatively small amount compared to the uptake by the tumour when injected intratumourally) on account of its proliferant vascularity. However, it is also noted that the mice, both prostate-cancer bearing mice and normal mice, appeared to be healthy in outward appearance behaviour and movement, although no formal testing of cognitive function etc. was undertaken as part of this study. Locally, intratumorally injected nanoparticles with concentrations ranging from a few mg ml^−1^ to a few hundreds of mg ml^−1^ have been studied in the several in vivo experiments which are available in the literature [19]. More importantly, several studies have been shown cytotoxic analysis of iron particles in mice, rats and humans and demonstrating mild to tolerable side effects up to concentrations of 4,000 mg Fe kg^−1^ [20]. In a prospective phase I trial of locally recurrent prostate cancer patients, Johannsen et al. studied the morbidity and quality of life during thermotherapy after intraprostatic injection of iron oxide NPs. The authors showed that iron oxide NP deposits were detectable in the prostate one year after the thermal therapy but interestingly no systemic toxicity was observed at a median follow-up at 17.5 months. The authors concluded that interstitial heating using magnetic nanoparticles was feasible and well tolerated in patients with locally recurrent prostate cancer [21].

Given the results presented in this study, we feel it would be necessary to optimise the time for a future clinical application of magnetic coil heating in relation to the time at which the FDG-mNP is injected to minimise the heating of FDG-mNP taken up by normal tissues and to ensure the dissipation of FDG-mNP into normal tissues.This will be the subject of further research, involving intravenous and intratumoural applications.

We also note that there is a need for optimisation of the concentration of FDG-mNP used in the treatment of different cancer types and this will be the subject of further research. Our next step will involve injecting the FDG-mNPs (a) intratumourally into the prostate gland of a mouse together with the application of magnetic hyperthemic heating to the prostate gland of the mouse. Although the direct intratumoural injection of FDG-mNPs has obvious advantages (e.g., with a local injection, the applied magnetic hyperthermia could likewise be targeted locally to a given area of the body containing the cancer) the intravenous route of application also gives other potential advantages relating to treatment. In instances where cancer has metastasized to other areas of the body, the intravenous route avails the FDG-mNPs to wider uptake by widely distributed cancer cells. In this way, widespread cancer cells (even those too small to be detected using current imaging techniques e.g. PET, MRI) could readily uptake the FDG-mNPs, such that magnetic hyperthermia could then be applied whole body, thus potentially leading to the destruction of these widespread metastases.

The results of the previous in vitro studies and the results of this current in vivo study presented in this paper will be used to further research of FDG-mNPs by applying hyperthermia to mice injected with FDG-mNPs at the concentrations indicated in this current in vivo pilot study. We note that our quantitative results regarding the uptake of FDG-mNPs by tissue (including the prostate tumour) are based on % of iron uptake by the tissue, and that the data obtained in this pilot study have been based on results obtained from a small number of mice. However, we nevertheless feel that this approach concerning the uptake of FDG-mNPs in cancer cells (and the subsequent application of magnetic heating to the FDG-mNPs taken up by cancer cells) is worthy of further investigation.

## Conclusions

The results of our in vivo pilot study involving the uptake of FDG-mNPs by cancerous tissue and normal tissue in mice indicate that:

➢ The FDG-mNPs are rapidly taken up (both by the intravenous route and when directly injected into the tumour) by the cancer cells in all three groups of cancerous tissue in mice.
➢ That little FDG-mNPs are taken up by non-cancerous tissues.
➢ That over time the concentration of FDF-mNPs in both cancerous and non-cancerous tissue appears to decrease over time.
➢ That there are no observable detrimental effects in the mice injected with the FDG-mNPs over the times periods used in our study.
➢ That our results are worthy of further study, and that this technique of injecting FDG-mNPs into cells—directly or by intravenous injection coupled with hyperthermic techniques (magnetic heating coil application)—may produce an effective method of cancer cell destruction, which could potentially have clinical applications.

## Abbreviations

FDG: fludeoxyglucose
FOV: field of view
H&E: Haemotoxylin and Eosin
ICG: indocyanine green
ID: inner diameter
mNPs: magnetic nanoparticles
MRI: Magnetic Resonance Imaging
MGE: Multiple Gradient Echo
PBS: phosphate buffer saline
PET: Positron Emission Tomography
RF: radiofrequency
ROI: region of interest
TE: echo time
TR: repetition time
